# A diet enriched in omega-3 PUFA and inulin prevents type 1 diabetes by restoring gut barrier integrity and immune homeostasis in NOD mice

**DOI:** 10.3389/fimmu.2022.1089987

**Published:** 2023-01-13

**Authors:** Marta Lo Conte, Martina Antonini Cencicchio, Marynka Ulaszewska, Angelica Nobili, Ilaria Cosorich, Roberto Ferrarese, Luca Massimino, Annapaola Andolfo, Federica Ungaro, Nicasio Mancini, Marika Falcone

**Affiliations:** ^1^ Autoimmune Pathogenesis Unit, Division of Immunology, Transplantation and Infectious Diseases, IRCCS San Raffaele Scientific Institute, Milan, Italy; ^2^ Università Vita-Salute San Raffaele, Milan, Italy; ^3^ Proteomics and Metabolomics Facility (ProMeFa), IRCCS San Raffaele Scientific Institute, Milan, Italy; ^4^ Laboratory of Medical Microbiology and Virology, IRCCS San Raffaele Scientific Institute, Milan, Italy; ^5^ Experimental Gastroenterology Unit, Division of Immunology, Transplantation and Infectious Diseases, IRCCS San Raffaele Scientific Institute, Milan, Italy; ^6^ Laboratory of Medical Microbiology and Virology, Università “Vita-Salute” San Raffaele, Milan, Italy

**Keywords:** anti-inflammatory diet (AID), Type 1 diabetes, gut barrier integrity, mucus layer, gut microbiota, regulatory T cells

## Abstract

**Introduction:**

The integrity of the gut barrier (GB) is fundamental to regulate the crosstalk between the microbiota and the immune system and to prevent inflammation and autoimmunity at the intestinal level but also in organs distal from the gut such as the pancreatic islets. In support to this idea, we recently demonstrated that breakage of GB integrity leads to activation of islet-reactive T cells and triggers autoimmune Type 1 Diabetes (T1D). In T1D patients as in the NOD mice, the spontaneous model of autoimmune diabetes, there are alterations of the GB that specifically affect structure and composition of the mucus layer; however, it is yet to be determined whether a causal link between breakage of the GB integrity and occurrence of autoimmune T1D exists.

**Methods:**

Here we restored GB integrity in the NOD mice through administration of an anti-inflammatory diet (AID- enriched in soluble fiber inulin and omega 3-PUFA) and tested the effect on T1D pathogenesis.

**Results:**

We found that the AID prevented T1D in NOD mice by restoring GB integrity with increased mucus layer thickness and higher mRNA transcripts of structural (Muc2) and immunoregulatory mucins (Muc1 and Muc3) as well as of tight junction proteins (claudin1). Restoration of GB integrity was linked to reduction of intestinal inflammation (i.e., reduced expression of IL-1β, IL-23 and IL-17 transcripts) and expansion of regulatory T cells (FoxP3^+^ Treg cells and IL-10^+^ Tr1 cells) at the expenses of effector Th1/Th17 cells in the intestine, pancreatic lymph nodes (PLN) and intra-islet lymphocytes (IIL) of AID-fed NOD mice. Importantly, the restoration of GB integrity and immune homeostasis were associated with enhanced concentrations of anti-inflammatory metabolites of the ω3/ω6 polyunsaturated fatty acids (PUFA) and arachidonic pathways and modifications of the microbiome profile with increased relative abundance of mucus-modulating bacterial species such as *Akkermansia muciniphila* and *Akkermansia glycaniphila*.

**Discussion:**

Our data provide evidence that the restoration of GB integrity and intestinal immune homeostasis through administration of a tolerogenic AID that changed the gut microbial and metabolic profiles prevents autoimmune T1D in preclinical models.

## Graphical Abstract

An anti-inflammatory diet enriched in fibers (inulin) and omega3 is capable to increased relative abundance of protective gut bacteria and thickness of the mucus layer in non-obese diabetic (NOD) mice. As consequence, gut immune homeostasis is restored with an expansion of regulatory T cell subsets (FoxP3+ Treg and IL-10+Tr1 cells) in the intestinal mucosa but also in pancreatic lymph nodes and islets where they may limit aggressiveness of islet-reactive T cells thus inducing protection from autoimmune T1D.

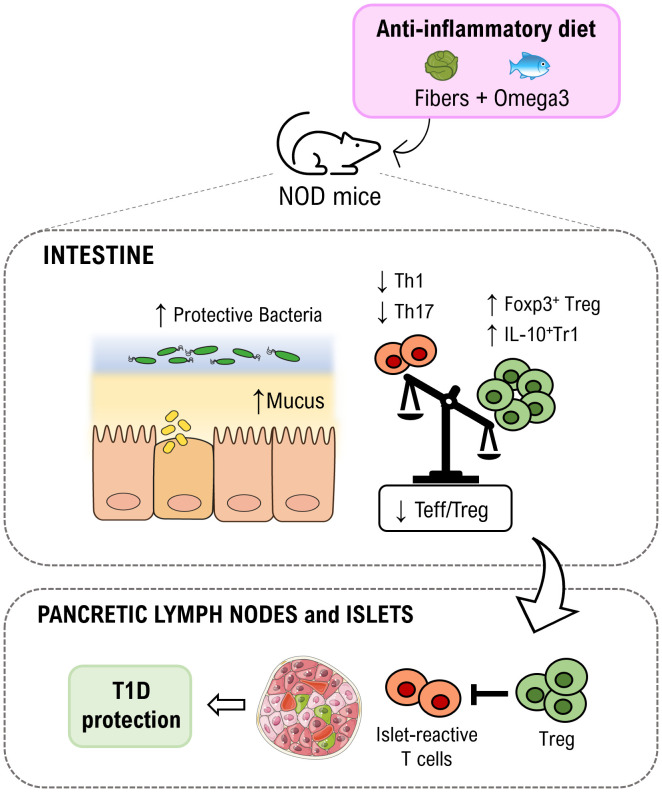

## Introduction

T1D is a multifactorial autoimmune disease characterized by autoimmune destruction of insulin-producing beta cells of pancreatic islets of Langerhans (1). The numerous genetic and environmental factors that regulate T1D pathogenesis are still largely unknown. Recent evidence indicates that the gut environment is fundamental to modulate autoimmunity at sites distal from the intestine including the pancreatic islets in T1D (2). The microbiota composition, intestinal inflammation and breakage of GB integrity are all important players in modulating the effector phenotype and aggressiveness of self(islet)-reactive T cells and for triggering the early events of autoimmune T1D (3–5). In particular, the integrity of the GB composed of the epithelial barrier and the mucus layer is crucial to prevent beta cell autoimmunity and T1D. In support to this view, alterations of the GB integrity and of the mucus layer are found in humans and preclinical models of T1D (e.g., the NOD mice) and precede the clinical signs of disease (5–10). Furthermore, we recently found that low-grade intestinal inflammation and GB damage in TCR transgenic *BDC2.5NOD* mice carrying a large islet-reactive T cell repertoire triggers activation of the diabetogenic T cells and T1D (10). A causal link between GB alterations and T1D pathogenesis and the mechanisms through which GB damage leads to activation of beta cell autoimmunity are yet to be determined. The GB is an important physical barrier that avoids uncontrolled passage of microbial components into the gut mucosa and systemic circulation (11). Changes to the protective mucus layer, the body’s first line of defense at the mucosal epithelia, can alter exposure of the underlying epithelium to foreign antigens and favor activation of self(islet)-reactive T cells (10, 12). Moreover, the GB and, specifically, the mucus layer contain important immune regulatory factors such as mucins and anti-microbial peptides (AMP) that are essential to maintain immune homeostasis and induce Treg cell differentiation at the intestinal and systemic level thus suppressing inflammatory/effector T cells (13, 14), possibly including self(islet)-reactive T cells.

The diet composition is fundamental to maintain gut immune homeostasis and GB integrity and to prevent inflammation (15). Dietary factors provoke or protect from intestinal inflammation and breakage of GB integrity both directly and through microbiota modulation (16–19). Some dietary substances such as saturated fatty acids compromise the GB and allow passage of luminal contents (food antigens, microbiota components) into the mucosal and submucosal layers in proximity to immune cells (20). This results in activation of immune cells, inflammation and further damage of GB integrity. Other dietary components such as soluble fibers and PUFA play a beneficial role on GB integrity. For example, dietary administration of omega-3 PUFA maintains immune tolerance in the gut by directly suppressing inflammation and promoting a beneficial anti-inflammatory microbiota profile (21, 22). The direct anti-inflammatory effect of omega-3 PUFA is related to block of release of proinflammatory eicosanoids and modulation of the functional phenotype of macrophages (23) with release of tolerogenic cytokines such as IL-10 and increased differentiation of regulatory T cells at the expenses of effector Th17 cells (24). The omega-3 PUFA effect on the microbiota profile includes a decrease of pro-inflammatory bacteria such as the Enterobacteriales with concomitant expansion of beneficial bifidobacteria and lactobacilli (25).

Nonfermentable fibers are another dietary factor that is fundamental to maintain gut immune tolerance and prevent inflammation. Dietary fibers are neither digested nor absorbed in the intestine but they critically modulate gut microbiota composition by favoring growth of bifidobacteria and lactobacilli that degrade fibers into SCFAs (butyrate, propionate and acetate), tolerogenic metabolites promoting FoxP3 Treg cell differentiation and preventing intestinal inflammation (26), and/or directly secrete anti-inflammatory metabolites such as the lactic acid (27). Importantly, dietary fibers also have direct microbiota-independent beneficial effects. For example, they trigger release of tolerogenic cytokine IL-10 by dendritic cells (28) and directly promote GB integrity acting *via* modulation of epithelial tight junction proteins (29), goblet cell function (30), and glycocalyx maturation (31). Fibers such as inulin-type fructans induce changes of the intestinal mucosa characterized by higher villi, deeper crypts, increased number of Goblet cells (GC), and increased production of mucins resulting in a thicker mucus layer on the colonic epithelium (32–34). Moreover, inulin promotes formation of the epithelial glycocalyx that is crucial to regulate bacterial-epithelial crosstalk and to support GB function (31).

In order to demonstrate that the GB alterations found in the spontaneous preclinical model of T1D, the NOD mice, are mechanistically linked to T1D pathogenesis we exploited a dietary approach, an anti-inflammatory diet (AID) enriched with omega-3 PUFA and inulin, to restore GB integrity and immune homeostasis in NOD mice and test the effect on T1D pathogenesis.

## Materials and methods

### Mice

Female NOD and BALB/c mice were purchased from Charles River Laboratories. All mice were maintained under specific pathogen-free conditions in the animal facility at San Raffaele Scientific Institute and all experiments were conducted in accordance with the Institutional Animal Care and Use Committee (IACUC) according with the rules of the Italian Ministry of Health.

### Dietary regimens

Immediately after weaning, 4-weeks old NOD mice were separated in two groups, housed in different cages and fed with either an anti-inflammatory diet (AID) or standard (STD) diet. The AID was designed with the supervision of a nutritionist and purchased from Rieper S.p.A. (Altromin C1000). The AID diet was enriched with fish oil (3,6%), linseed oil (2%) and corn oil (7,7%), for a total of 13,3% of fats (1:3 PUFA ω3:ω6 ratio with specifically 20g/kg ω3 and 60g/kg ω6). The STD diet contained only 5% of fats (100% Corn oil) with 1:190 PUFA ω3:ω6 ratio (0,15g/kg ω3 and 28,5g/kg ω6). AID diet contained 8% of fibers (100% Inulin) while the STD diet with only 5% of fibers (100% cellulose). For more information about composition of the AID see **Supplementary Table S1**.

### Diabetes incidence

Diabetes was monitored by weekly measurements of blood glucose levels with a GB35 Ascensia Breeze 2 glucometer (Bayer). The animals were considered diabetic after two consecutive blood glucose measurements > 250mg/dl according to standard method (35).

### *In vivo* gut permeability assay

Intestinal permeability was determined by FITC-dextran assay as previously described (36). Briefly, 20 mL/kg of body weight of PBS containing 25 mg/mL FITC-conjugated dextran (FITC-dextran; molecular mass, 4.4 kDa; FD4, Sigma-Aldrich) was administered to each mouse by oral gavage. After 4 h, blood was collected and the concentration of fluorescein was determined by spectrophotofluorometry (Wallac Victor; Perkin-Elmer Life Sciences) with an excitation wavelength of 485 nm and an emission wavelength of 530 nm using serially diluted samples of the FITC-dextran marker as standard.

### Cell isolation

Intestinal mononuclear cells were isolated from the small and large intestinal lamina propria as previously described (Lefrancois and Lycke, 2001; Weigmann et al., 2007). Briefly, after the removal of Peyer’s patches, small and large intestines were flushed with PBS, opened longitudinally, and incubated twice with 5 mM EDTA and 1 mM DTT for 20 min at 37°C to remove epithelial cells and adipose tissue. Then, the intestines were cut into small pieces and digested in HBSS containing 0.5 mg/ml Collagenase D (Roche Diagnostics), 1 mg/ml Dispase II (Roche Diagnostics) and 5 U/ml DNase I (Sigma-Aldrich) for 20 min at 37°C in a shaking incubator. The digested tissues were washed, resuspended in 5 ml of 40% Percoll (Sigma-Aldrich) and overlaid on 2.5 ml of 80% Percoll in a 15-ml Falcon tube. Percoll gradient separation was performed by centrifugation at 1000 g for 20 min at 20°C. The interface cells were collected and used as intestinal lymphocytes for FACS analysis. Splenocytes and pancreatic lymph node (PLN) cells were isolated by meshing of the tissues. For isolation of intra-islet lymphocytes (IIL), freshly collected pancreata were cut into small pieces with a scissor and digested 3 times for 15min at 37°C with agitation in 3 ml/pancreas of Hank’s Balanced Salt Solution (HBSS) 1X with Ca2^+^ and Mg2^+^ complemented with 1 mg/ml of Collagenase IV (Gibco). The digestion was stopped by extensive washing with HBSS containing 5% FBS and 5-minutes incubation on ice to let clumps of undigested pancreatic tissue to settle. Intra-islet lymphocytes (IIL) were collected from the supernatants of the three digestion/incubation cycles and extensively washed in HBSS 5% FBS before use in FACS analysis.

### Flow cytometry

Single cell suspensions from different organs were resuspended in staining buffer containing PBS, 1% FBS and 0.09% NaN_3_ and stained with monoclonal antibodies against surface markers. For intracellular cytokine staining, single cell suspensions isolated from different organs were stimulated for 2.5 hours with the Leukocyte Activation Cocktail (BD Bioscience). Cells were first stained for surface markers, then fixed and permeabilized using the BD Cytofix/Cytoperm kit (BD Bioscience), and finally stained for intracellular cytokines. The following antibodies were used: FITC anti-mouse CD3 (17A2, BD Biosciences), PercCP-cy5.5 anti-mouse CD4 (RM4-5, BD Biosciences), APC anti-mouse INF-γ (XMG1.2 BioLegend), APC-cy7 rat anti-mouse IL-17A (TC11-18H10. BD Bioscience), PE anti-mouse IL-10 (JES5-16E3 eBioscience), eFluor 647 anti-mouse Foxp3 (FJK-16s, eBiosciences) and APC anti-mouse CD25 (PC61, BioLegend). Dead cells were stained with Fixable Viability Dye eFluor 506 (eBioscience) and excluded from the analysis. Flow cytometry was performed using FACSCanto III (BD Biosciences) and data were analyzed with FCS Express V4 software (*De Novo* Software). See **Supplementary Figure 1** for gating strategy.

### RT-qPCR analysis

Immediately after sacrifice, mice’s colons were flushed with PBS, opened longitudinally and placed in 500μl of TRIzol reagent (Life Technologies). After homogenization with TissueRuptor (QIAGEN), RNA was extracted by adding 100μl of chloroform, precipitating the acqueous phase with 300μl of 70% ethanol and purifying RNA with RNeasy Mini Kit (QIAGEN). RNA was retrotranscribed with SuperScript III First-Strand Synthesis System following manufacturer’s instructions (Life Technologies). Real time qPCR assay was performed with SYBR Select Master Mix (Life Technologies) using primers specific for different tight junction proteins, mucins and cytokines (**Supplementary Table S2**) on a ViiA 7 Real-Time PCR System (Life Technologies). Transcript expression was normalized against Rpl32 gene (housekeeping gene). See **Supplementary Table 2** for the list of primers used in this study.

### Histology

Segments of the distal colon were fixed in Carnoy’s fixative (60% dry methanol, 30% chloroform, and 10% acetic acid), washed in absolute methanol, ethanol, xylene, embedded in paraffin wax and sectioned at 6 μm. For mucus layer structure evaluation, colon sections were stained with Alcian Blue staining kit, pH 2.5 (Sigma-Aldrich) and images acquired with Axio Imager M2m Light Microscope (ZEISS). The proportion of GC in colon sections was determined within the total number of cells in randomly selected stained sections. To assess histopathological signs of diabetes, pancreata were fixed in formalin, embedded in paraffin, sectioned into 4-μm slices and then stained with hematoxylin–eosin for insulitis score.

### 16S rRNA microbiota analysis

Total DNA was isolated from the mucosa and luminal content of the large intestine of NOD mice using PowerFecalTM DNA Isolation kit (MoBio) following the manufacturers’ instructions. Microbiome characterization was performed by amplification of three regions of the 16S rRNA (V3, V4, V5) using universal primer pairs. The analysis of metabolically active microbiota was performed by pyrosequencing of rRNA cDNA 16S (GS Junior, Roche Diagnostics GmbH). Sequences with a high-quality score were used for the taxonomic analysis with QIIME (Quantitative Insights into Microbial Ecology version 1.6).

### Metabolomic analysis

Fresh snap-frozen feces were collected from mice after 10 weeks of diet and used for metabolomics analysis. Metabolites were extracted three times using the following mixture of solvent, acetonitrile:methanol:water = 4:4:2. The collected supernatants were filtered on 96 well-plate with positive pressure manifold (0.22 μm pore size), dried under gentle stream of nitrogen and recomposed with 100 µl of water containing 0.1% formic acid. A QC sample was prepared by taking 20 µL from each sample. The samples were analysed by LC-MS/MS in 5 technical replicates by injecting 3 µl on the TripleTOF 5600+ mass spectrometer (SCIEX) connected to the UPLC 1290 (Agilent Technologies). The chromatographic separation occurred on a Waters Acquity UPLC HSS T3 column (100x2.1 mm, 1.8 µm) through the following 25 minutes gradient of solvent A (water containing 0.1% formic acid) and solvent B (acetonitrile containing 0.1% formic acid) at flow rate of 600 µl/min: 1 min of equilibration at 2% B; from 2% B to 95% B in 14 min; washing step at 95% B for 5 min, re-equilibration at 2% B for 5 min. The mass spectrometry analysis was performed both in positive and negative polarity, in the range of m/z 50-500 (TOF-MS scan with an accumulation time of 0.15 sec), with a SWATH acquisition comprising 10 windows of 45 Da each (accumulation time of 0.07 sec). The MS-DIAL software version 4.7.0 was used to analyze MS and MS/MS data for the identification of metabolites.

### Statistical analysis

Cumulative diabetes incidence was calculated using the Kaplan-Meier estimation, whereas statistical significance was evaluated by the log-rank test. Statistical significance of the differences between 2 or more samples for qRT-PCR data and immunological data was calculated by unpaired 2-tailed Student’s *t*-test or ANOVA respectively. Metabolic profile data, normalized to feces weight, were submitted to ANOVA test, with FDR correction. We could observe 17630 features in positive polarity and 10535 features in negative polarity with FDR p-value < 0.05. The PCA analysis was performed using an in-house developed R script, while the heatmap was obtained using the MeV software v. 4.9.0 (2). For statistical analysis of the microbiome, microbial reads were discriminated against human reads with BMTagger (ftp.ncbi.nlm.nih.gov/pub/agarwala/bmtagger/) and mapped to the collection of all available genomes (https://www.ncbi.nlm.nih.gov/genome/) with Kraken2 for exact alignment of k-mers and accurate read classification (37). Relative abundance profiling and differential analysis were performed with DESeq2 upon variance-stabilizing transformation (38). Visualization was performed with ggplot2 (39). Species alpha diversity and dominance indices were calculated with vegan (https://cran.r-project.org/web/packages/vegan). *p* values <0.05 were considered statistically significant.

## Results

### Administration of AID prevent autoimmune diabetes in NOD mice

**Figure 1:**
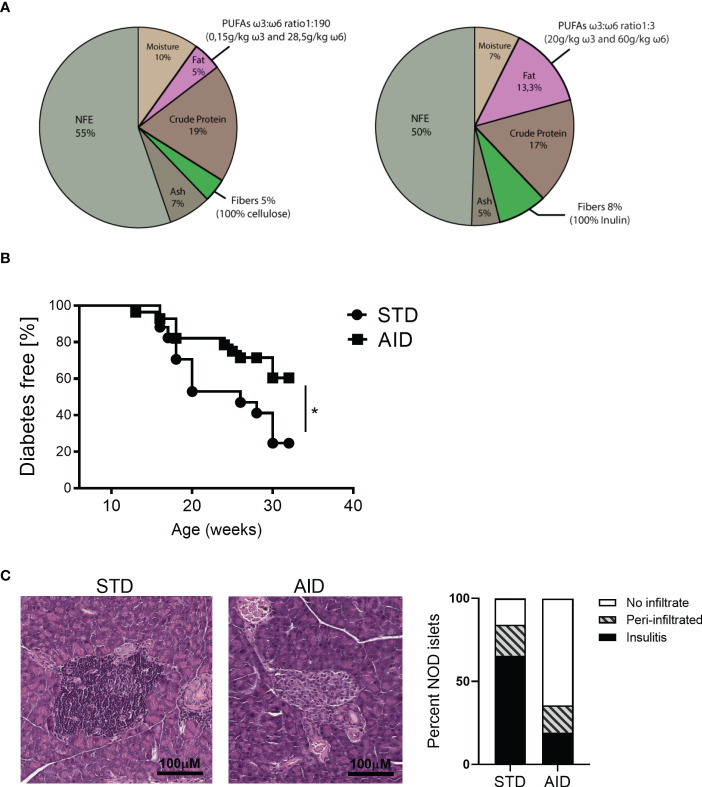
NOD mice fed with anti-inflammatory diet were protected from autoimmune diabetes. **(A)** 4-weeks-old female NOD mice were fed with anti-inflammatory diet (AID) or standard (STD) diet up to 32 weeks of age (end of the experiment). In the AID the total amount of fats was 13,3% (3,6% of fish oil, 2% of linseed oil and 7% of corn oil), with PUFA ω3:ω6 ratio of 1:3 (20000mg/kg ω3 and 60000mg/kg ω6), and 8% of fibers (100% Inulin). STD diet contained 5% of fats (100% corn oil) with PUFA ω3:ω6 ratio of 1:190 (150 mg/kg ω3 and 28500 mg/kg ω6) and 5% of fibers (100% Cellulose). NFE, nitrogen-free extract **(B)** Incidence of autoimmune diabetes in NOD mice fed with AID (n=25) and STD diet (n=20). **(C)** Diabetic mice from each group (AID or STD diet-fed) were sacrificed after two consecutive measurements of hyperglycemia (> 250mg/dl), while diabetes resistant mice were sacrificed at the end of the experiment (32 weeks of age). Hematoxylin and eosin staining of pancreatic tissues was performed to detect lymphocyte infiltrates. On the left: one representative image of pancreatic tissue showing islet infiltration (in a diabetic NOD mouse fed with STD diet) or intact islets (in a diabetes-resistant NOD mice fed with AID diet) are shown. On the right: percentages of pancreatic islets infiltrated, peri-infiltrated with lymphocytes or intact (no infiltrates) out of total analyzed islets in Hematoxylin and Eosin-stained pancreata of NOD mice fed with AID or STD diet. Five randomly selected sections were analyzed from each mouse (n=6 mice per group). *p < 0.05.

NOD mice that spontaneously develop autoimmune diabetes have a defect of the GB that primarily affects the mucus layer structure and composition (10), however a causal relationship between loss of gut barrier integrity and autoimmunity in this preclinical model of T1D is yet to be determined. Here, we restored GB integrity in NOD mice and test the effect on T1D occurrence. We decided to use a dietary approach containing nutrients known to reduce intestinal inflammation and promote GB function. To this aim, we elaborated a specific anti-inflammatory diet (AID) that includes omega-3 PUFA capable to reduce gut inflammation and promote immune tolerance, i.e., FoxP3^+^ Treg and IL-10^+^Tr1 cell differentiation (21–24), and inulin, a soluble fiber, that plays a direct beneficial effect on the GB and mucus layer integrity and promotes FoxP3^+^ Treg cell differentiation (26, 28–31) (**Figure 1A** and **Supplementary Table S1** for diet composition). Female NOD mice were either fed with standard (STD) diet or with the AID starting at 4 weeks of age just after weaning and before onset of beta cell autoimmunity and first signs of GB damage occur (10). In our animal facility female NOD mice normally develop spontaneous autoimmune diabetes with an incidence of 80-90% between 16 and 30 weeks of age. Our data reveal that the administration of the AID protected female NOD mice from occurrence of T1D (**Figure 1B**) with 40% of diabetic mice in the AID-fed group compared to 80% in the STD diet-fed counterparts at 32 weeks of age (end of experiment) (*p< 0.05*). The protective effect of the AID was confirmed at the histological level and NOD mice fed with the AID showed reduced degree of insulitis and increased number of intact islets (70%) compared to STD diet-fed mice (15%) (**Figure 1C**).

### AID restored GB integrity by promoting expression of tight junction proteins and mucins

**Figure 2:**
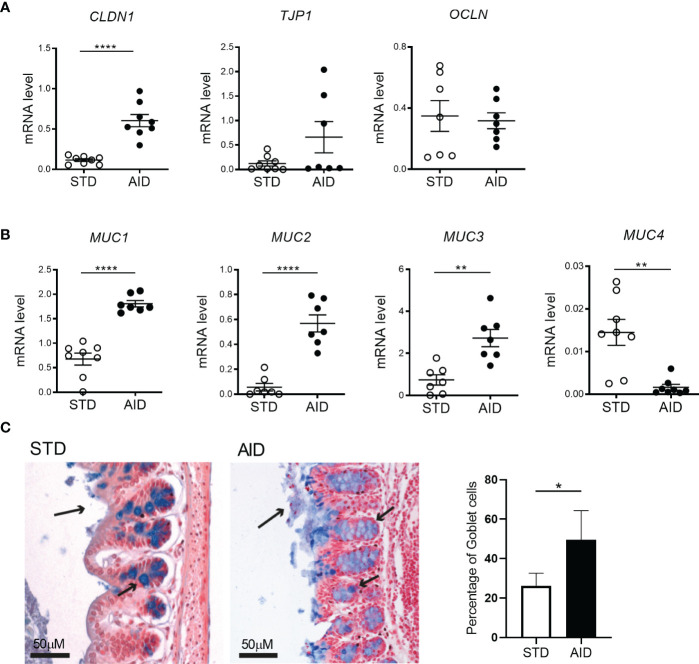
AID restores gut barrier integrity in NOD mice. **(A)** RT-qPCR analysis of claudin 1 (Cldn1), tight junction protein 1 (Tjp1) and Occludin (Ocln) on tissue homogenates from the intestine of NOD mice fed with anti-inflammatory (AID) or standard (STD) diet at 14 weeks of age (n=8 per group). **(B)** RT-qPCR analysis of Muc1, Muc2, Muc3, and Muc4 mucin genes in the intestine of NOD mice fed with AID or STD diet (n=8 per group). **(C)** Alcian Blue staining of colon sections (20X) of NOD mice fed with AID or STD diet. The arrows on the apical side of the intestine (long arrow) indicate the thickness of the mucus layer while the smaller arrows along the crypts point to GC (stained in Blue). The percentage of GC in the two groups (right panel) was calculated within the total number of epithelial cells on randomly selected sections (n=5 per group, 6 sections/mouse). All data are presented as mean ± SEM. *p < 0.05; **p < 0.01; ****p < 0.0001.

The GB alterations in the NOD mice are detectable at 10-12 weeks of age concomitantly with onset of beta cell autoimmunity and thus suggesting that they may have a triggering effect on T1D pathogenesis (10). In order to assess whether prevention of T1D in AID-fed NOD mice is linked to restoration of GB integrity, we performed a gut permeability test (FITC-dextran) and measured by RT-qPCR the expression of mRNA transcripts of tight junction proteins and analyzed different biomarkers of mucus layer integrity (mucus layer thickness, percentage of GC, mRNA expression levels of mucins) in the intestine of AID vs STD diet-fed NOD mice. Our FITC-dextran test confirmed previous findings of increased gut permeability in NOD mice compared to non-autoimmune mice (**Supplementary Figure 2**) and a decrease of gut permeability in AID-fed NOD mice compared to STD diet-fed counterparts at 24 weeks of age but not earlier in the pre-diabetic phase (14 weeks of age) (**Supplementary Figure 2**). Importantly, we observed that AID-fed NOD mice had increased mRNA expression levels of claudin 1 (CLDN1), one structural protein of the intestinal epithelial barrier (IEB) (**Figure 2A**; *p<0.0001*). Importantly, mRNA transcripts relatives to several mucins including the mucus structural mucin Muc2 (*p<0.0001*) and immune-regulatory mucins Muc1 (*p<0.0001*) and Muc3 (*p<0.01*) were also up-regulated in the intestinal tissue of AID-fed NOD mice (**Figure 2B**), while Muc4, a pro-inflammatory mucin (40), is decreased in AID-fed NOD mice (**Figure 2B**; *p<0.01*). Restoration of the mucus layer integrity in AID-fed NOD mice was confirmed at the histological level with an almost complete absence of mucus layer in STD-fed NOD mice that was corrected by administration of AID (**Figure 2C**). In line with this observation, we measured a significantly higher percentage of GC in the large intestine of AID-fed NOD mice compared to their STD diet-fed counterparts (**Figure 2C**; *p<0.05*).

### AID promoted differentiation/expansion of regulatory FoxP3^+^ Treg and IL-10^+^Tr1 cells

**Figure 3:**
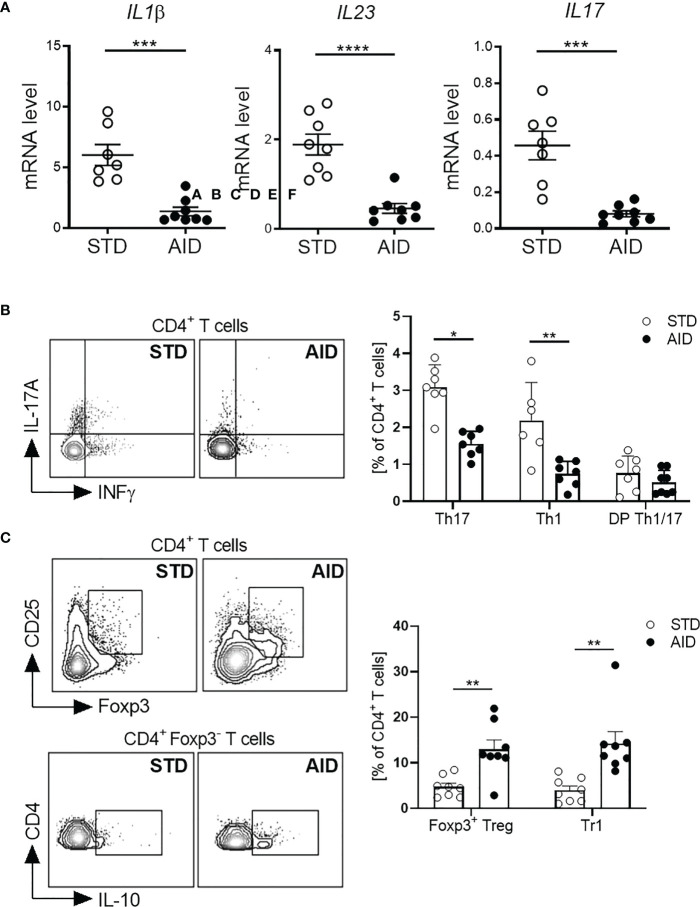
AID reduces intestinal inflammation and promotes gut immune homeostasis in NOD mice. **(A)** RT-qPCR analysis of cytokine genes encoding interleukin-1β (Il1b), subunit p19 of IL-23 (Il23), and IL-17A (Il17a) on tissue homogenates from the intestine of NOD mice fed with anti-inflammatory (AID) or standard (STD) diet at 14 weeks of age (n=8 per group). **(B)** Representative flow cytometry plots (*Left*) and percentages (*Right*) of INF-γ^+^CD4^+^ (Th1 cells), IL-17^+^CD4^+^ (Th17 cells) and INF-γ^+^IL-17^+^CD4^+^ (DP Th1/17 cells) cells out of total CD4^+^ T cells in the small intestinal tissue of 12-week-old NOD mice fed with AID or STD diet (n=7-8 mice/group). **(C)** Representative flow cytometry plots (*Left*) and percentages (*Right*) of FoxP3^+^CD25^+^CD4^+^ (FoxP3^+^Treg cells) and IL-10^+^FOXP3^-^CD4^+^ (Tr1 cells) cells out of total CD4^+^ T cells in the small intestinal lamina propria of 12-week-old NOD mice fed with AID or STD diet (n=7-8 mice/group). All data are presented as mean percentages ± SEM. *p < 0.05; **p < 0.01.

**Figure 4:**
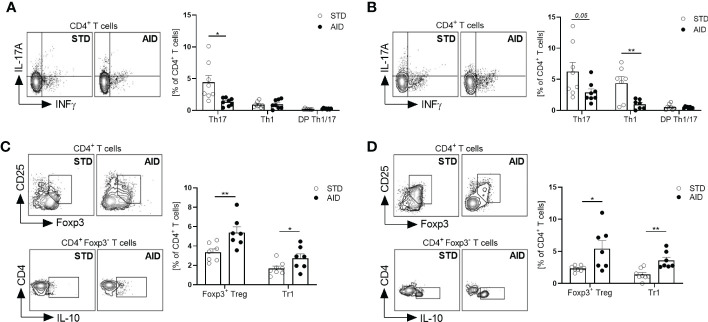
AID reduces the Teff/Treg cell ratio in the pancreatic lymph nodes and islets of NOD mice. **(A, B)**: Representative flow cytometry plots (*Left*) and percentage (*Right*) of INFγ+CD4+(Th1), IL-17+CD4+ (Th17) and INF-γ^+^IL-17^+^CD4^+^ (DP Th1/17 cells) cells out of total CD4+ T cells in pancreatic lymph nodes (PLN) **(A)** and pancreatic intra-islet lymphocytes (IIL) **(B)** of 12-week-old NOD mice fed with AID or STD diet (n=8 per group). **(C, D)** Representative flow cytometry plots (*Left*) and percentage (*Right*) of FoxP3^+^CD25^+^CD4^+^ (FoxP3^+^Treg cells) and IL-10^+^FOXP3^-^CD4^+^ (Tr1 cells) cells out of total CD4^+^ T cells in the PLN **(C)** and pancreatic IIL **(D)** of 12-week-old NOD mice fed with AID or STD diet (n=8 per group). All data are presented as mean percentages ± SEM. *p < 0.05; **p < 0.01.

Next, we asked whether restoration of the GB integrity (IEB and mucus layer) and protection from T1D in AID-fed NOD mice were associated with reduction of inflammation and induction of tolerogenic mechanisms in the gut mucosa. Specifically, since T1D occurrence is associated with acquisition of an effector phenotype by diabetogenic T cells in the gut (10), we asked whether T1D protection in AID-fed NOD mice was linked to a shift of functional phenotype of intestinal T cells from an effector Th17/Th1 type to a regulatory type (FoxP3^+^ Treg and IL-10^+^ Tr1 cells). First, we analyzed gut inflammation by measuring mRNA expression levels of inflammatory cytokines IL-1β, IL-23 and IL-17 in the intestinal tissues and found a statistically significant reduction of those inflammatory cytokines in AID-fed NOD mice (**Figure 3A**; *p<0.001* for IL-1β and IL-17 and *p<0.0001* for IL-23). Furthermore, our FACS analysis of the functional phenotype of intestinal T cells revealed that AID feeding in NOD mice significantly reduced the relative percentages of effector Th1 and Th17 particularly in the small intestine (**Figure 3B**; *p<0.05* for Th17 and *p<0.01* for Th1 cells) and to a lesser extent in the large intestine (**Supplementary Figure 3**) while simultaneously promoting expansion/differentiation of FoxP3^+^ Treg cells in the small and large intestine (**Figure 3C**; *p<0.01* and **Supplementary Figure 3**) and IL-10^+^ Tr1 cells in the small intestine (**Figure 3C**; *p<0.01*). Importantly, improved immune tolerance mechanisms, i.e., increased FoxP3^+^ Treg and IL-10^+^ Tr1 cells, and decrease of Teff cell differentiation found in the intestinal mucosa of AID-fed mice were spread to pancreatic lymph nodes (PLN) and islets. In fact, we detected a decrease of effector Th17 cells in the PLN (**Figure 4A**; p<0.05) and, most importantly, a decrease of effector Th17 and Th1 cells within the intra-islet lymphocytes (IIL) of AID-fed NOD mice (**Figure 4B**; *p=0.05* for Th17 cells and *p<0.01* for Th1 cells). Simultaneously, we measured an increase of the relative percentages of both FoxP3^+^ Treg cells and IL-10^+^ Tr1 cells in the PLN (**Figure 4C**; *p<0.01* for FoxP3^+^Treg cells and *p<0.05* for Tr1 cells) and IIL (**Figure 4D**; *p<0.05* for FoxP3^+^Treg cells and *p<0.01* for Tr1 cells) of AID-fed NOD mice compared to their STD diet-fed counterparts.

### AID modified the microbial and metabolomic profile in the intestine of NOD mice

**Figure 5:**
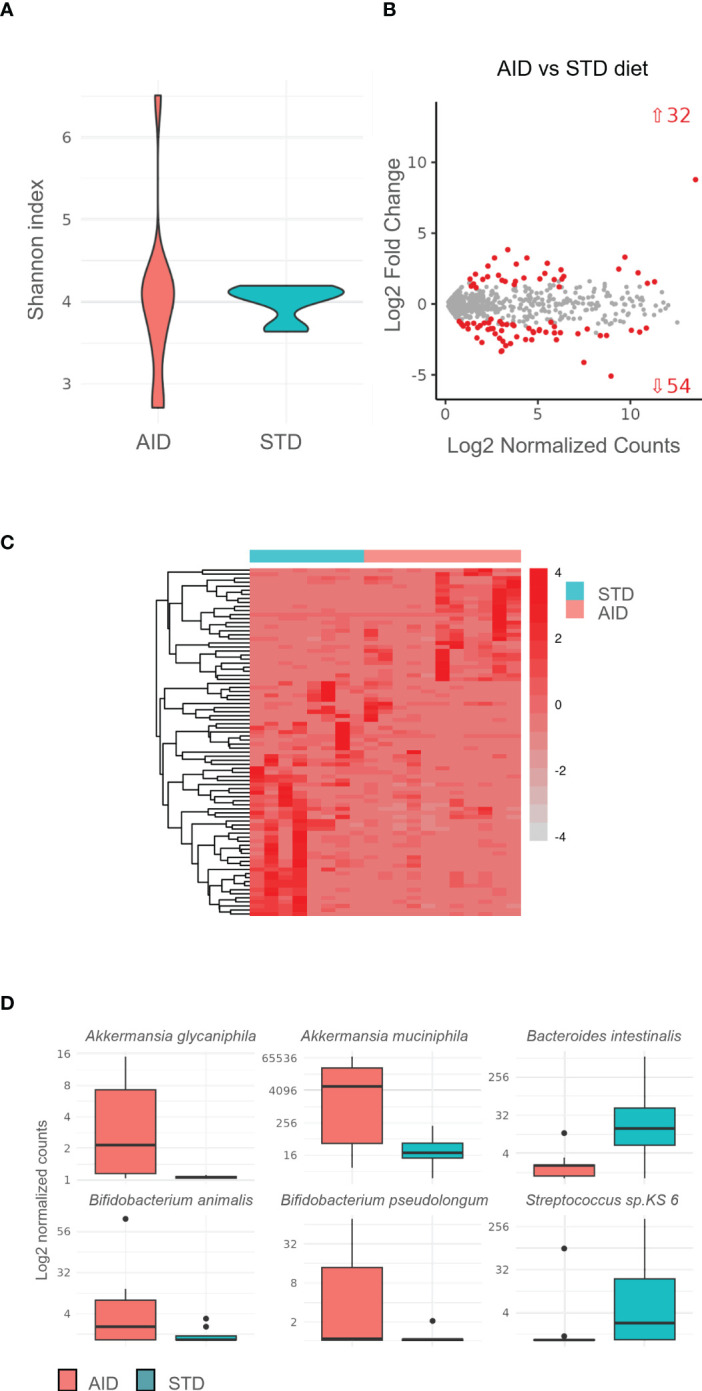
Gut microbiota profiles of AID and STD-fed NOD mice. Microbiota isolated from luminal content and brushed intestinal mucosa of AID or STD-fed NOD mice at 12 weeks of age (n=6 per group) was assessed by 16S Amplicon Sequencinq of DNA and analysed with QIIME software. **(A)** alpha-diversity in AID vs STD-diet fed NOD mice based on Shannon index. **(B)** Differentially expressed bacterial species between AID and STD diet fed NOD mice. **(C)** Heatmap of selected differentially represented bacterial species in AID vs STD-diet fed NOD mice. **(D)** Relative abundance of selected differentially represented bacterial species in AID vs STD-fed NOD mice.

**Figure 6:**
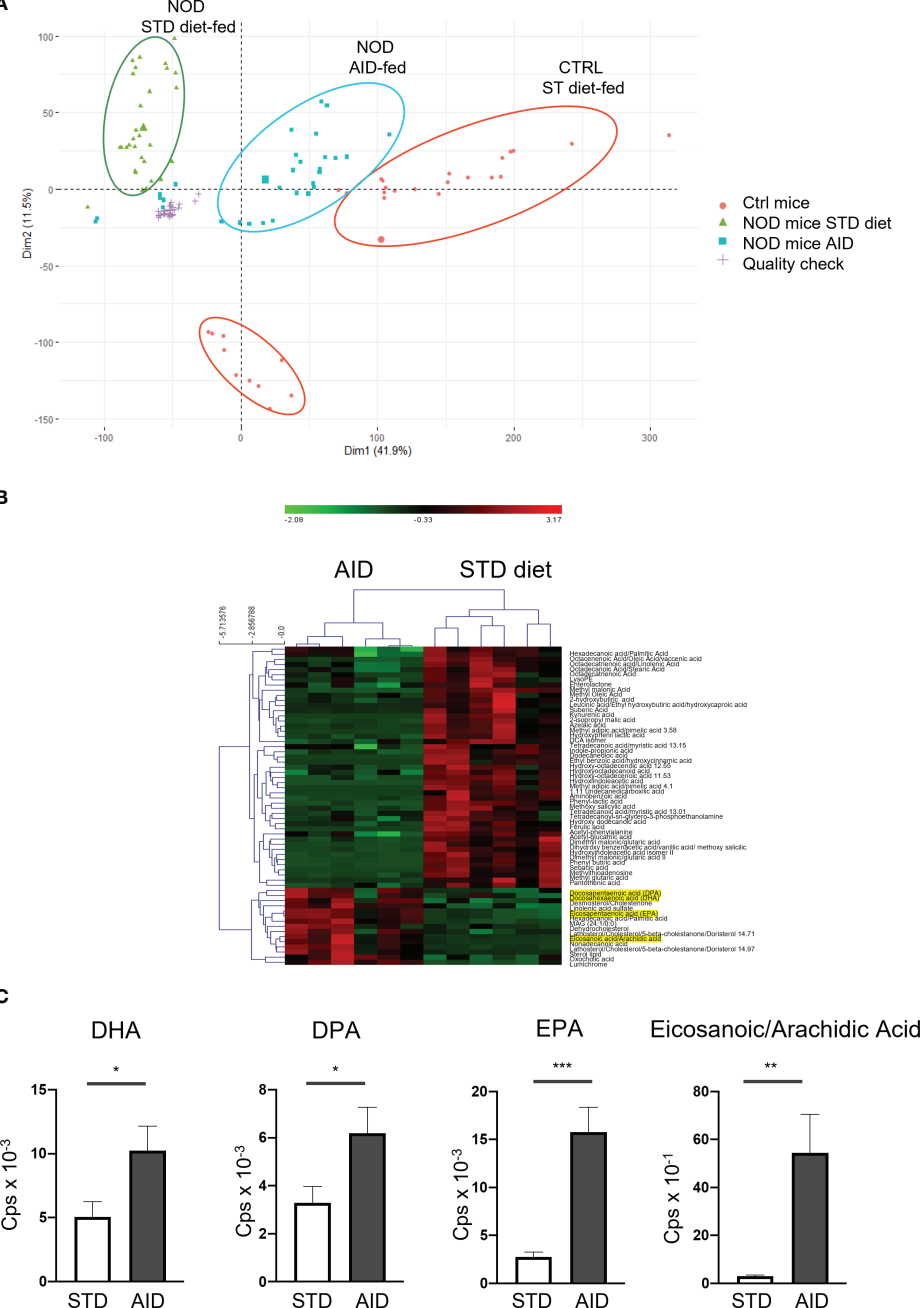
Metabolomic profiles of stools from AID or STD diet-fed NOD mice. **(A)** Representative PCA analysis of 17630 features in positive polarity with FDR *p-value < 0.05* in STD-diet and AID-fed NOD mice and non-autoimmune Balb/c mice (n=6 per group). Technical replicates for the QC sample clustered together, showing a very reproducible LC-MS/MS analysis. The larger symbol per group indicates the mean of all the samples. **(B)** Heatmap of the peak areas for the 62 annotated metabolites which were significantly different between STD-fed vs AID-fed NOD mice. The overrepresented metabolites are in red, while the underrepresented ones are indicated in green. 15 out of 62 metabolites were upregulated in the group of AID-fed NOD mice. **(C)** Selected differentially represented metabolites in AID vs STD-fed NOD mice. Data are expressed as mean percentages ± SEM. *p < 0.05; **p < 0.01; ***p < 0.001.

To clarify the mechanisms responsible for restoration of GB integrity and immune homeostasis in AID-fed NOD mice, we analyzed the microbial and the metabolic profile at the intestinal level. Our 16s rRNA analysis of the gut microbiota profiles (from the intestinal mucosa and luminal content) showed important modifications in AID-fed NOD mice compared to STD diet-fed controls. Specifically, we detected an increased alpha-diversity (Shannon index) in the AID-fed NOD mice (**Figure 5A**), a characteristic of the gut microbiota profile that is normally associated with reduced intestinal inflammation and protection from T1D. We also measured statistically significant differences in the relative abundance of 86 bacterial taxa (**Figures 5B, C** and **Supplementary Table 3**). Notably, we found a very high increase in AID-fed NOD mice of the relative abundance of two *Akkermansia* species (log2fold change of 8.7 for *Akkermansia muciniphila* and 2.1 for *Akkermansia glycaniphila*) (**Figure 5D** and **Supplementary Table 3**; *p=6.9X10^-14^ and p<0.01* respectively), two bacterial species associated with mucus-regeneration (41–44). We also detected a statistically significant augment of relative abundance of beneficial species previously linked with dietary administration of omega-3 PUFA and soluble fibers such as bifidobacteria (*Bifidobacterium pseudolongum* and *Bifidobacterium animalis*) *(* 21, 22, 26 *):* (**Figure 5D** and **Supplementary Table 3**; *p=4X10^-5^* and *p<0.05* respectively). Conversely, some pro-inflammatory bacterial strains such as *Bacteroides intestinalis* and *Streptococcus* sp. KS 6 were decreased in AID-fed NOD mice (**Figure 5D** and **Supplementary Table 3**; *p=4X10^-4^* and *p<0.01* respectively). Next, we asked whether the AID modified the metabolic environment in the intestine of NOD mice. Our untargeted metabolic analysis with high performance liquid chromatography tandem-mass spectrometry (LC-MS/MS) revealed a completely different metabolic profile in AID-fed NOD mice compared to NOD mice fed with STD diet (**Figure 6A**). Importantly, the metabolic profile of AID-fed NOD mice was much closer to that of a control non-autoimmune mouse strain (Balb/c mice) (**Figure 6A**), even if the Balb/c mice clustered in two groups possibly due to different environmental conditions (**Figure 6A**). Numerous metabolites were differentially represented in NOD mice fed with AID compared with STD diet (**Figure 6B**) including several metabolites involved in the ω6/ω3 PUFA metabolic pathway that perform pro-resolving actions in experimental models of colitis such as Docosapentaenoic acid (DPA), Docesahexaenoic acid (DHA) and Eicosapentaenoic acid (EPA) (45–47) and in the arachidonic acid metabolic pathway (Eicosanoic acid/Arachidic acid) involved in regulation of mucosal immunity and gut epithelial barrier function (48) (**Figures 6B, C** and **Supplementary Table 4**; *p<0.05* for DHA and DPA, *p<0.001* for EPA and *p<0.01* for Eicosanoic/Arachidic acid).

## Discussion

Previous reports indicated that specific diet regimens such as an omega-3 PUFA-enriched diet or a diet supplemented with tolerogenic metabolites (acetate, propionate) prevent T1D in humans and preclinical models (49–51). Those dietary regimens counter-regulate T1D possibly by promoting growth of beneficial bacteria and dampening intestinal inflammation (51, 52), however an association between dietary protection from T1D and restoration of GB integrity was never demonstrated. Here we specifically designed an anti-inflammatory diet aimed at restoring GB integrity and mucus layer structure in NOD mice. Our data showing protection from T1D in AID-fed NOD mice with restoration of GB integrity provide proof-of-concept that an inflammatory gut environment and GB damage with modifications of mucus layer structure and composition play a causal role in the autoimmune pathogenesis of T1D in the NOD mice.

Recent evidence indicates that damage of the GB plays a pathogenic role in T1D. In line with this idea, several signs of intestinal inflammation with increased intestinal permeability, GB damage associated with lymphocyte infiltration and presence of pro-inflammatory cytokines are present in patients and preclinical models of T1D (5–10). The observation that breakage of GB integrity with subsequent increased antigen trafficking and occurrence of low-grade intestinal inflammation precede the onset of T1D suggests that GB damage is directly related to autoimmune pathogenesis rather than secondary to diabetes-induced metabolic alterations (5, 10). A causal link between loss of GB integrity and occurrence of T1D was demonstrated by the finding that induction of low-grade intestinal inflammation in TCR transgenic *BDC2.5NOD* mice with an expanded self(islet)-reactive T cell repertoire promoted intestinal activation of the diabetogenic T cells thus triggering autoimmune diabetes (10). Damage of the GB integrity with alterations of the mucus structure and composition are found in the NOD mice, the spontaneous preclinical model of T1D (10), however there is yet no evidence that those defects are not an epiphenomenon but rather play a direct pathogenic role in T1D occurrence. Here, we demonstrated that restoring GB integrity in NOD mice through administration of an AID that specifically increased expression of tight junction proteins and mucins thus restoring a normal mucus layer architecture plays a beneficial role and prevented T1D. How does the GB damage provoke activation of beta cell autoimmunity and T1D occurrence? The GB is an important gatekeeper that regulates the interaction between the gut commensal microbiota and the immune system. The GB maintains the physical separation between microbial species and immune cells residing in the gut mucosa but also plays important immune regulatory functions. Specifically, the mucus layer contain immune-regulatory molecules such as Muc1 and Muc3 that are crucial to maintain immune tolerance towards bacterial antigens and prevent inflammation (53). Here, we did not detect an amelioration of the gut permeability in the prediabetic phase in AID-fed mice but only after the onset of diabetes in the STD-fed group, thus indicating that restoration of physical separation between gut mucosa and the intestinal lumen is not crucial to prevent beta cell autoimmunity in AID-fed NOD mice. Conversely, we observed an increase of mucus structural protein and immune regulatory mucins that preceded occurrence of beta cell autoimmunity (14 weeks of age) and thus could play a direct beneficial effect on dampening the autoimmune process in T1D. In fact, in the NOD mice the GB damage mostly relates to defects of mucus layer structure and composition leading to loss of immune homeostasis and inflammation with a predominance of effector Th1/Th17 cells and a defect of regulatory T cells in the gut (10). Our data demonstrate that those mucus layer alterations and defective gut immune homeostasis are directly linked to T1D pathogenesis in NOD mice. In fact, in AID-fed NOD mice, restoration of GB integrity with increased structural mucus layer component Muc2 and immune-regulatory mucins (Muc1 and Muc3) and subsequent increase of FoxP3^+^ Treg and IL-10^+^ Tr1 cells prevented T1D. The increased relative percentages of FoxP3^+^Treg/Tr1 cells and decrease of effector T cells (Th1/Th17) was not limited to the gut mucosa but a shift of the functional phenotype of T cells from a pro-inflammatory Th1/Th17 type to a protective Treg phenotype (FoxP3^+^ Treg and IL-10^+^Tr1) was also detected in the draining PLN and intra-islets lymphocyte (IIL) infiltrates of AID-fed NOD mice. Recent evidence in different preclinical models of T1D indicates that self-(islet)-reactive T cells are activated at the intestinal level, possibly through microbiota-induced mechanisms of molecular mimicry (54, 55) and acquire effector phenotype (Th1/Th17) (10). Hence, we speculate that a tolerogenic gut environment induced by AID can shift the functional phenotype of self(islet)-reactive T cells from an effector Th1/Th17 type towards a regulatory FoxP3^+^ and/or Tr1 cell type at the intestinal level, thus counter-regulating T1D pathogenesis.

How does the AID restore GB integrity in the NOD mice? Alterations of the gut commensal microbiota composition have been reported in patients and preclinical models of T1D (4, 56–58). Diet is one of the most effective measures to modulate the composition of the commensal gut microbiota (18). Previous reports indicate that diets enriched in omega-3 PUFA or inulin fibers promote a beneficial gut microbiota composition promoting overgrowth of anti-inflammatory bifidobacteria and lactobacilli (21, 22, 26). Here, we found that our AID enriched in omega-3 PUFA and inulin had a strong impact on the gut microbiota composition of NOD mice. First, in T1D-protected AID-fed NOD mice we detected a higher overall diversity (alpha diversity based on Shannon index), a condition previously associated with a decreased risk to develop clinical T1D in humans (3). In addition, we found an increased relative abundance of beneficial species previous associated with omega-3 PUFA and inulin such as bifidobacteria (*Bifidobacterium pseudolongum* and *Bifidobacterium animalis*). However, the most significant modification that we observed in the gut microbiota composition of AID-fed NOD mice was an 8-fold increase in the relative abundance of *Akkermansia muciniphila* and a 2-fold increase in *Akkermansia glycaniphila*. Those are mucus-degrading bacteria belonging to the Verrucomicrobia phylum that produce a wide array of mucin degrading enzymes fundamental to promote mucus regeneration, increase mucus layer thickness and maintain its function of physical and biological barrier (41–44). Importantly, the levels of *A. muciniphila* inversely correlate with intestinal inflammation (59) and previous reports associated the presence of *Akkermansia muciniphila* in the commensal gut microbiota with diabetes protection in NOD mice (60, 61). The absence of this mucus-degrading strain in a specific NOD mouse colony (NOD/Jax mice) was linked with high diabetes incidence, while transferring of *A. muciniphila* to those NOD mice either through oral gavage or co-housing with a diabetes-resistant NOD mouse colony (NOD/MrkTac) prevented T1D. The protective effect of *A. muciniphila* was related to enhanced mucus production, restoration of physical GB integrity and immune homeostasis with increased number of regulatory FoxP3^+^ Treg cells and transcripts levels of Treg-associated cytokines IL-10 and TGF-β in PLN and islets (61). In accordance with those findings, in our AID-fed NOD mice T1D protection was associated with high increase in *A. muciniphila*, restoration of the mucus layer architecture with augmented mRNA expression of structural (Muc2) and immune-regulatory mucins (Muc1 and Muc3) and enhanced FoxP3^+^ Treg and IL-10^+^ Tr1 cells in the PLN and IIL.

Another important mechanism through which the AID could counter-regulate T1D is through modulation of the intestinal metabolic profile. Recent evidence indicates that the metabolites that are present in the gut mucosa are important regulators of adaptive immunity at the intestinal level but also systemically (62). We found that AID-fed NOD mice have an activation of the ω3/ω6 PUFA metabolic pathway and arachidonic pathway that are associated with reduction of intestinal inflammation and restoration of GB integrity in different colitis models (45–48).

Diet composition is one of the strongest environmental factors affecting the composition of the gut microbiota but also the GB integrity and maintenance of immune homeostasis in the gut and systemically. Our data provide evidence that an AID designed to promote GB integrity and function is capable to prevent occurrence of T1D by improving mucus layer architecture and composition and promoting immune tolerance, i.e., FoxP3^+^ Treg and IL-10^+^ Tr1 expansion, in the intestine but also in PLN and islets of NOD mice. The intestinal environment is important for modulating T1D in preclinical models but also in humans affected by T1D. Commensal gut microbiota alterations, intestinal inflammation and GB damage are all factors that increase the risk to develop T1D in genetically susceptible individuals. Our results could pave the way to new dietary approaches aimed at restoring GB function and immune homeostasis with the final goal to prevent T1D in genetically “at-risk” children.

## Data availability statement

The datasets presented in this study can be found in online repositories. The name of the repository and accession number can be found below: NCBI Sequence Read Archive; PRJNA913009.

## Ethics statement

The animal study was reviewed and approved by the Institutional Animal Care and Use Committee (IACUC) of the IRCCS San Raffaele Scientific Institute according with the rules of the Italian Ministry of Health (IACUC #868).

## Author contributions

MLC and MAC performed *in vivo* experiments and FACS analysis. IC and AN designed the AID and analyzed data. RF and LM performed 16s rRNA and statistical analysis on microbiota profiles. MU and AA performed mass spectrometry and analyzed metabolomic data. MLC analyzed data and prepared figures. NM and FU contributed to design the study and to interpret data. MF served as principal investigator, analyzed and interpreted data, and wrote the manuscript. All authors contributed to the article and approved the submitted version.
