# Supplementary material for: A diet enriched in omega-3 PUFA and inulin prevents type 1 diabetes by restoring gut barrier integrity and immune homeostasis in NOD mice
Source: Front Immunol. 2023 Jan 13;13:1089987. doi: 10.3389/fimmu.2022.1089987 (PMC9880528; doi:10.3389/fimmu.2022.1089987)
Supplement: Supplementary file 1 [file DataSheet_1.pdf]

Supplementary Table 1

Ingredient	gr/kg	Ingredient	gr/kg
Crude protein	172,335	Zinc	0,025
Crude fat	113,587	Copper	0,0048
Crude fibre	1,461	Iodine	0,0004
Crude Ash	46,417	Molybdenum	0,0001
Moisture	76,430	Fluorine	0,0034
Disaccharide(s)	118,4	Selenium	0,0002
Polynsaccharide(s)	415,63	Cobalt	0,0001
Metab. Energy	4,070	Capric acid C-10:0	0,077
Lysine	17,391	Lauric acid C-12:0	0,113
Methionine	7,216	Myristic acid C-14:0	2,613
Cystine	3,189	Pentadecanoic acid C-15:0	0,221
Threonine	7,142	Palmitic acid C-16:0	1,731
Tryptophan	1,974	Palmitoleic acid C-16:1	3,389
Arginine	9,813	Margaric acid	0,077
Histidine	5,266	Stearic acid C-18:0	4,728
Isoleucine	7,210	Oleic acid C-18:1	24
Phenylalanine	7,156	Linoleic acid C-18:2	49,243
Valine	3,281	Linolenic acid C-18:3	9,495
Alanine	2,502	Arachidic acid C-20:0	0,873
Aspartic acid	3,561	Eicosaenoic acid C-20:1	1,680
Glutamic acid	23,615	Eicosadienoic acid C-20:2	0,729
Glycine	3,123	Arachidonic acid C-20:4	0,545
Proline	12,733	Eicosapentaenoic acid C-20:5	7,493
Serine	5,251	Behenic acid C-22:0	0,498
Tyrosine	9,271	Acetic acid C-2:0	0,077
Vitamin A	15	Docosahexaenoic acid C-22:6	2,777
Vitamin D3	0,5	Ricosanic acid	0,077
Vitamin E	0,183	Nervonic acid C-24:1	0,077
Vitamin K3	0,01	Erucic acid C-22:1	1,008
Vitamin B1	0,023	Aluminium	0,0239
Vitamin B2	0,023		
Vitamin B6	0,015		
Vitamin B12	0,0004		
Nicotinic acid	0,501		
Pantothenic acid	0,504		
Folic acid	0,1		
Biotin	0,002		
Choline Chloride	1,011		
Inositol	1,11		
Vitamin C	0,2		
Calcium	7,782		
Phosphorus	6,522		
Digest. Phosphorus	6,250		
Magnesium	0,570		
Sodium	2,070		
Potassium	5,908		
Sulfur	2,657		
Chlorine	3,025		
Iron	0,148		
Manganese	0,084		

Table S1: Composition of the anti-inflammatory diet (AID). AID diet was designed by a dietitian. It was enriched with fish oil (3,6%), linseed oil (2%) and corn oil (7,7%), for a total of 13,3% of fats (PUFAs ω3:ω6 ratio 1:3 with specifically 20000mg/kg ω3 and 60000mg/kg ω6) and 8% of fiber (100% Inulin).

Supplementary Figure 1

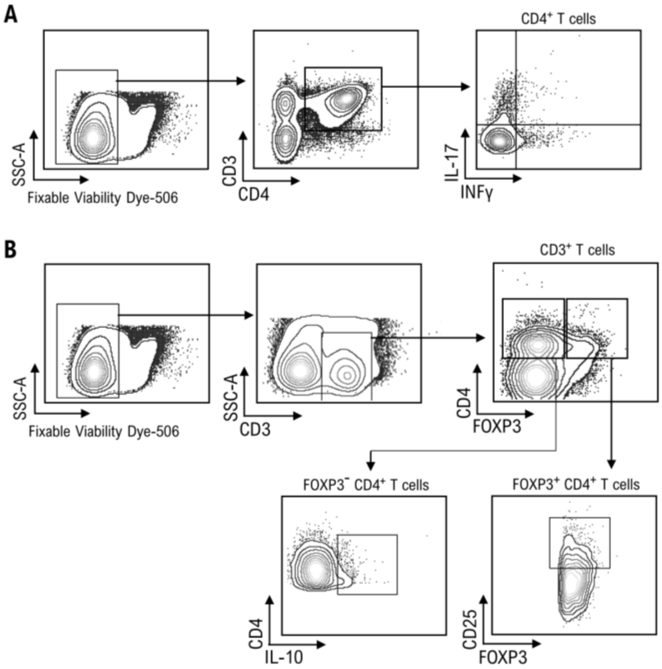

Figure S1: **A.** Flow cytometry gating strategy used to define the following T helper cells subsets used in the study: Th1 cells (Dye-506$^+$CD3$^+$CD4$^+$INF-γ^+), Th17 cells (Dye-506$^+$CD3$^+$CD4$^+$IL-17$^+$) and double positive IL-17$^+$ INF-γ^+ T cells (Dye-506$^+$CD3$^+$CD4$^+$INF-γ^+ IL-17A$^+$). **B.** Flow cytometry gating strategy used to define the following T regulatory cells subsets used in the study: FOXP3$^+$ regulatory T cells (Dye-506$^+$CD3$^+$CD4$^+$FOXP3$^+$ CD25$^+$) and type 1 regulatory T cells (Dye506$^+$CD3$^+$CD4$^+$FOXP3$^-$IL-10$^+$).

Supplementary Table 2

Target	*Forward primer*	*Reverse primer*
Il1b	CAA CCA ACA AGT GAT ATT CTC CAT G	GAT CCA CAC TCT CCA GCT GCA
Il17a	TCA TCC CTC AAA GCT CAG CG	TTC ATT GCG GTG GAG AGT CC
Il23	GTG ACA TGT GGG TTG AGC CT	GGC ATG AGG TTC CGA AAA GC
Muc1	TAC CCT ACC TAC CAC ACT CAC G	CTG CTA CTG CCA TTA CCT GC
Muc2	CAC CAA CAC GTC AAA AAT CG	GGT CTC TCG ATC ACC ACC AT
Muc3	CTT CCA GCC TTC CCT AAA CC	TCC ACA GAT CCA TGC AAA AC
Muc4	GAG AGT TCC CTG GCT GTG TC	GGA CAT GGG TGT CTG TGT TG
Rpl32	AAG CGA AAC TGG CGG AAA C	TAA CCG ATG TTG GGC ATC AG
Tjp1	ACC CGA AAC TGA TGC TGT GGA TAG	AAA TGG CCG GGC AGA ACT TGT GTA
Cldn1	GAT GTG GAT GGC TGT CAT TG	CCT GGC CAA ATT CAT ACC TG
Ocln	TCC GTG AGG CCT TTT GAA	GGT GCA TAA TGA TTG GGT TTG

Table S2: List of primers' sequences used in the study.

Supplementary Figure 2

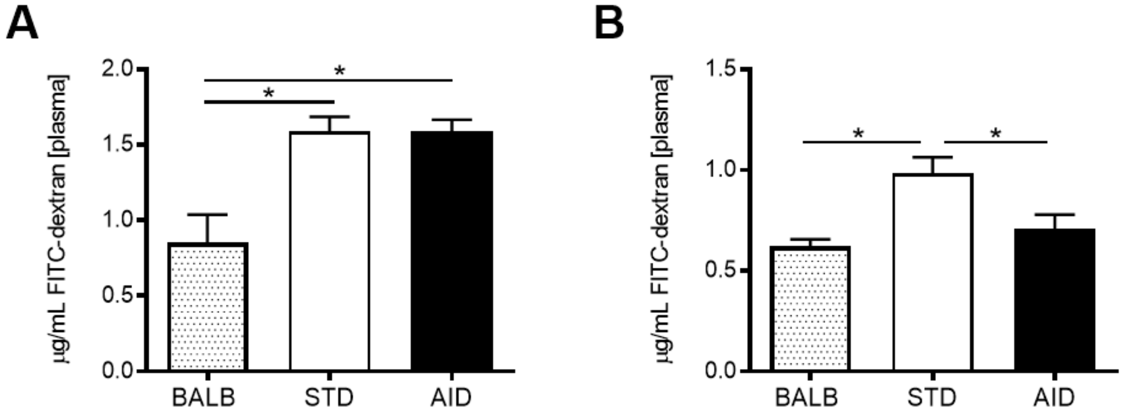

Figure S2. Gut permeability test in AID and STD diet-fed NOD mice and non-autoimmune mice. A-B. FITC-dextran in vivo permeability assay in female NOD mice fed with AID or STD diet at 14 wees (**A**) and 24 weeks of age (**B**) of dietary regimen; age-matched female BALB/c mice were used as control mice. All data are presented as mean percentages ± SEM. *p<0.05.

Supplementary Figure 3

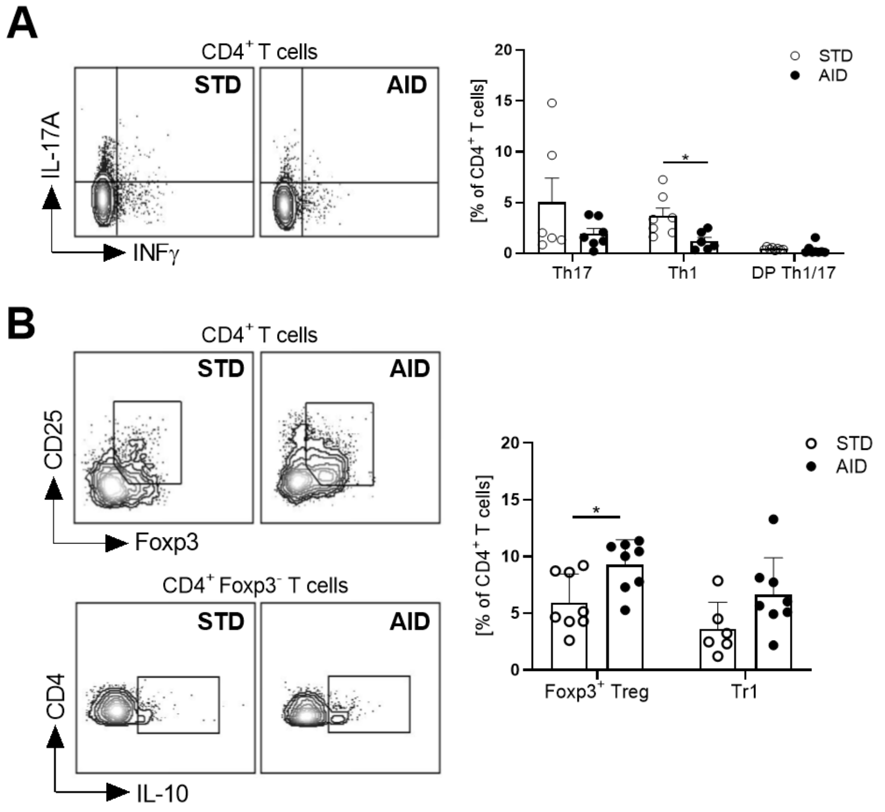

Figure S3: Immunological profiles in the intestine of AID or STD diet-fed NOD mice. A. Representative flow cytometry plots (*Left*) and percentages (*Right*) of INF-γ^+CD4$^+$ (Th1), IL-17$^+$CD4$^+$ (Th17) and INF-γ^+IL-17$^+$CD4$^+$ (DP Th1/17) cells out of total CD4$^+$ T cells in the large intestinal lamina propria of 12-week-old NOD mice fed with AID or STD diet starting at weaning (4 weeks of age). **B.** Representative flow cytometry plots (*Left*) and percentage (*Right*) of FoxP3$^+$CD25$^+$CD4$^+$ (Treg) and IL-10$^+$FOXP3$^-$CD4$^+$ (Tr1) cells out of total CD4$^+$ T cells in the large intestinal lamina propria of 12-week-old NOD mice fed with AID or STD diet. All data are presented as mean percentages ± SEM (n=7-8 mice/group). *$p<0.05$.

Supplementary Table 3

Gene (species)	base mean	Log2FoldChange	lfcSE	stat	p value	padj
Akkermansia muciniphila	11784,8553	8,780185628	1,17228108	7,489829683	6,90E-14	3,40E-11
Alistipes communis	496,3541918	-5,089713289	0,95197856	-5,346457893	8,97E-08	2,21E-05
Alistipes dispar	178,9693137	-4,122542987	0,84634803	-4,870978417	1,11E-06	1,82E-04
Alistipes onderdonkii	8,282397228	-3,307741477	0,719846755	-4,595063402	4,33E-06	5,33E-04
Lactobacillus acetotolerans	8,054354284	-3,352334418	0,76664419	-4,372738306	1,23E-05	0,0012098
Persephonella marina	6,281793499	3,250774534	0,786505832	4,133185545	3,58E-05	0,002933309
Bifidobacterium pseudolongum	10,33232292	3,834285827	0,93561749	4,098133979	4,16E-05	0,002933309
Larkinella sp. IE-0392	9,564201708	-2,941320869	0,726079589	-4,050962063	5,10E-05	0,003143336
Streptococcus sp. KS 6	3,912115882	-2,716563536	0,699900548	-3,881356492	1,04E-04	0,005690066
Desulfovibrio desulfuricans	14,22215055	2,815859333	0,739125146	3,809719302	1,39E-04	0,006858845
Hungatella hathewayi	21,16682833	3,250747469	0,86060171	3,777296085	1,59E-04	0,007105484
Sphingobacterium spiritivorum	8,607434376	-2,628759861	0,706791966	-3,719283733	2,00E-04	0,008207979
Orientia tsutsugamushi	3,256045901	-2,394515355	0,661024986	-3,622427904	2,92E-04	0,011067881
Vibrio sp. THAF191d	4,916659941	2,6799162	0,750562594	3,570543245	3,56E-04	0,012544795
Bacteroides sp. CBA7301	11,42973848	-2,796348826	0,789513955	-3,541861176	3,97E-04	0,013058404
Fimbriimonas ginsengisoli	14,47323292	-2,31344397	0,659839811	-3,506069093	4,55E-04	0,014012821
Phocaeicola vulgatus	834,4543808	3,306238425	0,948372936	3,486221824	4,90E-04	0,014206943
Treponema succinifaciens	6,448465942	-2,101620857	0,605597975	-3,470323453	5,20E-04	0,01423762
Desulfovibrio fairfieldensis	45,41622486	2,876698181	0,841591533	3,418164357	6,30E-04	0,014947095
Parvimonas micra	41,44194258	2,159065207	0,631869819	3,416946247	6,33E-04	0,014947095
Fructilactobacillus fructivorans	27,081356	-2,009635164	0,588389864	-3,415482296	6,37E-04	0,014947095
Brevefilum fermentans	33,50468278	-1,881704006	0,571404116	-3,293122944	9,91E-04	0,022203182
Alistipes sp. dk3624	331,8042269	-2,231633355	0,693064504	-3,21995044	0,001282128	0,027482126
Blautia argi	75,95481958	2,417141868	0,762904681	3,168340591	0,001533118	0,030347718
[Clostridium] innocuum	43,15054733	-1,987737981	0,627593043	-3,167240307	0,001538931	0,030347718
Runella sp. SP2	20,16858096	-2,508340847	0,801440305	-3,129791242	0,001749306	0,032408224
Duncaniella sp. C9	4,950019559	-1,800590073	0,57609243	-3,125522884	0,001774893	0,032408224
Bacteroides zoogleoformans	3,339429633	-1,663538732	0,535394961	-3,107124374	0,001889169	0,032504215
Thiospirochaeta perfilievii	7,606110161	-2,418279237	0,779263368	-3,103288742	0,001913828	0,032504215
Akkermansia glycaniphila	3,076720651	2,1034625	0,679956694	3,093524215	0,001977944	0,032504215
Bacteroides intestinalis	5,452824159	-1,74443152	0,577522337	-3,020543811	0,002523212	0,039703133
Sphingomonas ginsengisoli An et al. 2013	2,338206003	-1,760352635	0,584031273	-3,014141049	0,00257708	0,039703133
Porphyromonas crevioricanis	63,94565401	-2,523684478	0,851547973	-2,96364334	0,003040204	0,04541881
Bacteroides caecimuris	143,3265107	-2,098830038	0,713000998	-2,943656521	0,003243598	0,047032165
Thermobispora bispora	7,288669161	2,023310252	0,697874009	2,899248611	0,003740582	0,052688765
Phenylobacterium parvum	26,64997979	-1,95235156	0,679379556	-2,873727276	0,004056591	0,055506166
Parabacteroides goldsteinii	1368,474293	2,202312088	0,768607367	2,86532784	0,004165777	0,055506166
Porphyromonas cangingivalis	5,057145628	-1,332347357	0,471162304	-2,827788528	0,004687076	0,06080864
Altererythrobacter sp. B11	8,195086332	-1,881824441	0,667686145	-2,818426672	0,004825963	0,061005125
Anaerocolumna cellulosilytica	18,92017073	1,833313889	0,666911389	2,748961734	0,005978437	0,073680154
Coprococcus sp. ART55/1	33,87421044	-1,806925476	0,660558846	-2,735449668	0,006229511	0,073680154
Tolumonas auensis	4,8540601	1,929290934	0,705937489	2,732948685	0,006277011	0,073680154
Mesomycoplasma conjunctivae	3,64950578	-1,532222105	0,570679627	-2,684907666	0,007254985	0,083179242
Luteolibacter ambystomatis	2,513741547	1,758729666	0,658688646	2,670047034	0,007584062	0,083484464
Sulfuricella denitrificans	1,94688144	-1,564475317	0,58628688	-2,668446745	0,007620286	0,083484464
Butyrivibrio hungatei	22,85314901	-1,719909126	0,648174984	-2,653464214	0,007967022	0,08538569
Staphylococcus condimenti	13,76857317	1,599713008	0,60964929	2,623988964	0,00869066	0,091159477
Phoenicibacter congonensis	5,951731959	-1,736494427	0,667841658	-2,600158894	0,00931806	0,095704246
Ezakiella massiliensis	84,24074449	1,949177815	0,7553094	2,580634922	0,009861881	0,0992226
Parabacteroides distasonis	78,7472549	1,764801152	0,698132553	2,527888357	0,011475083	0,11170838
[Ruminococcus] gnavus	662,5505155	2,465522302	0,976282443	2,525419073	0,011556039	0,11170838
Oscillatoria acuminata	2,297179696	-1,390121398	0,551970557	-2,518470197	0,011786586	0,1117459
Sinimarinibacterium sp. NLF-5-8	4,047909567	1,738375688	0,700349941	2,482152972	0,01305912	0,121474456
Dialister pneumosintes	25,36169406	-2,523956902	1,020727341	-2,472704316	0,013409504	0,122290988
Limosilactobacillus reuteri	59,3167076	-1,599341951	0,648418356	-2,466527877	0,013643011	0,122290988
Bacteroides heparinolyticus	59,37197947	-2,042685049	0,838874756	-2,435029822	0,014890562	0,13109013
Helicobacter hepaticus	1,847923514	-1,443079484	0,595961333	-2,421431399	0,015459519	0,133711275
Legionella longbeachae	2,280488645	-1,388026089	0,579211488	-2,396406353	0,016556724	0,140732151
Bacteroides cellulosilyticus	1450,522124	-1,977476581	0,834260452	-2,370334799	0,017771984	0,148501494
Arachidicoccus sp. BS20	2,882975015	-1,358612854	0,581698554	-2,335596066	0,019512304	0,160326096
Bacteroides fragilis	1064,141109	-1,857632048	0,79914667	-2,324519538	0,020097666	0,162428675
Candidatus Fonsibacter ubiquis	1,807910498	-1,381530527	0,599825647	-2,303220168	0,021266459	0,169102647
Clostridium beijerinckii	8,418467217	-1,430707257	0,631386683	-2,265976297	0,023452835	0,183527737
Desulfoscipio gibsoniae	203,1988648	-1,765297144	0,783490509	-2,253118734	0,024251664	0,186813596
Bacteroides uniformis	1862,987391	-1,703794692	0,758214813	-2,247113434	0,024632779	0,186830151
Bifidobacterium animalis	2,860368635	1,418891103	0,638276367	2,223004291	0,026215511	0,19582192
Maribellus comscasis	4,273200136	-1,339400355	0,608555178	-2,200951373	0,027739466	0,204112791
Oleidesulfovibrio alaskensis	58,85763932	1,722132691	0,787575701	2,186624967	0,028769913	0,207713717
Thermomicrobium roseum	2,624130913	1,259932158	0,577284351	2,182515699	0,029071494	0,207713717
Rhodospirillum rubrum	8,327480736	1,673361627	0,775023326	2,159111308	0,030841533	0,217212508
Prevotella sp. oral taxon 299	1939,424372	1,461308156	0,679447894	2,150728803	0,031497612	0,218708768
Bulleidia sp. zg-1006	74,1102392	-2,026842931	0,946572122	-2,141245112	0,032254277	0,220852201
Alkalihalobacillus halodurans	12,35460298	-1,454212875	0,687574652	-2,114989071	0,034430879	0,232526346
Geosporobacter ferrireducens	5,512627391	-1,068719632	0,512157643	-2,086700543	0,036915212	0,243911141
Mageeibacillus indolicus	9,903357687	-1,454513548	0,697744239	-2,084594135	0,037106157	0,243911141
Sulfidibacter corallicola	34,40981477	1,772527642	0,857985067	2,065918988	0,03883613	0,250739409
Limosilactobacillus fermentum	13,00196477	-1,531461207	0,742534146	-2,062479168	0,039162139	0,250739409
Ureibacillus thermophilus	7,721300696	-1,23795735	0,603377004	-2,051714505	0,040197416	0,252095354
Tannerella serpentiformis	13,16162171	1,372598023	0,669667725	2,049670265	0,040396619	0,252095354
Campylobacter hominis	1,669814172	-1,228042473	0,602744671	-2,037417388	0,041608233	0,256410735
Prevotella sp. Rep29	419,539302	-2,225119115	1,101523181	-2,020038391	0,043379405	0,26402527
Halobacteroides halobius	70,93172597	1,210074403	0,601014627	2,013385946	0,044074043	0,264981746
Paroceanicella profunda	9,366286375	1,594725396	0,796730016	2,001588196	0,04532904	0,269243572
Clostridium estertheticum	52,03978404	-1,287893235	0,648876892	-1,984803666	0,047166319	0,276821375
[Clostridium] scindens	2539,45333	1,569341024	0,79887433	1,96444042	0,049479039	0,286315229
Desulfomonile tiedjei	3,154460325	1,160130605	0,591773293	1,960430827	0,049945456	0,286315229

Table S3: Bacterial species differentially represented in the stools of AID or STD diet-fed NOD mice. Microbiota isolated from luminal content and brushed intestinal mucosa of AID or STD-fed NOD mice at 12 weeks of age (n=6 per group) was assessed by 16S Amplicon Sequencinq of DNA and analysed with QIIME software.

Supplementary Table 4

Metabolite	Pathway	Immune regulatory role (pathway)
Docosapentaenoic acid (DPA)	ω6/ω3 PUFA	Anti-inflammatory in models of colitis
Docosahexaenoic acid (DHA)	ω6/ω3 PUFA	Anti-inflammatory in models of colitis
Eicosapentaenoic acid (EPA)	ω6/ω3 PUFA	Anti-inflammatory in models of colitis
Eicosanoic acid/Arachidic acid	Arachidonic acid	Regulation of mucosal immunity and epithelial gut barrier function

Table S4. List of metabolites increased in the intestine (stools) of AID-fed vs STD diet-fed NOD mice. The associated pathway and immune regulatory/anti-inflammatory function are indicated.
